# MYCN protein stability is a better prognostic indicator in neuroblastoma

**DOI:** 10.1186/s12887-022-03449-1

**Published:** 2022-07-11

**Authors:** Yi Yang, Jie Zhao, Yingwen Zhang, Tianyue Feng, Bo Yv, Jing Wang, Yijin Gao, Minzhi Yin, Jingyan Tang, Yanxin Li

**Affiliations:** 1 Pediatric Translational Medicine Institute, Department of Hematology & Oncology, Shanghai Children’s Medical Center, School of Medicine, Shanghai Jiao Tong University, National Health Committee Key Laboratory of Pediatric Hematology & Oncology, Shanghai, 200127 China; 2 Department of Hematology & Oncology, Shanghai Children’s Medical Center, School of Medicine, Shanghai Jiao Tong University, National Health Committee Key Laboratory of Pediatric Hematology & Oncology, Shanghai, 200127 China; 3 Gezhi Senior High School of Shanghai China, Shanghai, 200001 China; 4 State Key Laboratory of Oncogenes and Related Genes, Renji-Med X Clinical Stem Cell Research Center, Ren Ji Hospital, School of Medicine, Shanghai Jiao Tong University, Shanghai, 200127 China; 5 Department of general Surgery/Surgical Oncology Center, Shanghai Children’s Medical Center, Shanghai Jiao Tong University School of Medicine, Shanghai, 200127 China; 6 Department of Pathology, Shanghai Children’s Medical Center, Shanghai Jiao Tong University School of Medicine, Shanghai, 200127 China

**Keywords:** Neuroblastoma, MYCN, IHC, FISH, Protein stability

## Abstract

**Objective:**

*MYCN* oncogene amplification is associated with treatment failure and poor prognosis in neuroblastoma. To date, most detection methods of MYCN focus on DNA copy numbers instead of protein expression, which is the real one performing biological function, for poor antibodies. The current investigation was to explore a fast and reliable way to detect MYCN protein expression and evaluate its performance in predicting prognosis.

**Methods:**

Several MYCN antibodies were used to detect MYCN protein expression by immunohistochemistry (IHC), and one was chosen for further study. We correlated the IHC results of MYCN from 53 patients with *MYCN* fluorescence in situ hybridization (FISH) and identified the sensitivity and specificity of IHC. The relationship between patient prognosis and MYCN protein expression was detected from this foundation.

**Results:**

*MYCN* amplification status detected by FISH was most valuable for INSS stage 3 patients. In the cohort of 53 samples, IHC test demonstrated 80.0–85.7% concordance with FISH results. Further analyzing those cases with inconsistent results, we found that patients with *MYCN* amplification but low protein expression tumors always had a favorable prognosis. In contrast, if patients with *MYCN* non-amplified tumors were positive for MYCN protein, they had a poor prognosis.

**Conclusion:**

MYCN protein level is better than *MYCN* amplification status in predicting the prognosis of neuroblastoma patients. Joint of FISH and IHC could confirm MYCN protein stability and achieve better prediction effect than the singular method.

**Supplementary Information:**

The online version contains supplementary material available at 10.1186/s12887-022-03449-1.

## Highlights

The *MYCN* gene test is most valuable for INSS stage 3 patients to predict prognosis.

Compared to gene status, MYCN protein expression is more relevant to prognosis.

Combining FISH with IHC, MYCN protein stability could be identified.

**Supplementary Information:**

The online version contains supplementary material available at 10.1186/s12887-022-03449-1.

## Background

Neuroblastoma is the most common extracranial solid tumor in children [1, 2], and causes up to approximately 12% of pediatric cancer-related mortality [3]. *MYCN* oncogene amplification is a genetic marker detected in about 20–30% of neuroblastoma patients [4]. As a member of the *MYC* oncogene family, the overexpression of MYCN is closely correlated with high-grade malignancy, early distant metastasis, and poor clinical prognosis [5]. Even with increased intensity treatment, the five-year overall survival (OS) rate of patients with *MYCN* amplified tumors, independent of the risk stratification, is still less than that of patients with *MYCN* non-amplified tumors [6].

Since no reliable MYCN antibody is used in IHC, clinicians and researchers usually detect *MYCN* amplification status at the nucleic acid level. Conventional polymerase chain reaction (PCR) [7], quantitative real-time PCR (qPCR) [8, 9], semi-quantitative differential PCR (SQ-PCR) [10], droplet digital PCR (ddPCR) [11], FISH [12], chromogenic in situ hybridization (CISH) [13], and multiplex ligation-dependent probe amplification (MLPA) [14] are some common methods. The FISH result is an important index of risk stratification [15]. However, several studies have found that MYCN protein could be isolated from tumors without gene amplification, and tumors with *MYCN* amplification could not express protein [16–18]. For protein exerts the biological function [19], finding a rapid, reliable, and cost-effective strategy to detect MYCN protein expression is significant.

We compared the performance of several antibodies in IHC and finally chose one for further study in this research. Comparative analysis and survival analysis were performed to verify its feasibility in IHC. The correlation of MYCN protein expression with patient prognosis was another focus. Our results demonstrated that the antibody is reliable in IHC. Compared to gene status, MYCN protein expression and stability better predict outcomes.

## Methods

### Study population

A cohort of 53 neuroblastoma patients was selected as the main study object. They received curative surgery at Shanghai Children’s Medical Center (SCMC), Shanghai, China, between January 2010 and September 2019. 28 tumor samples of this cohort were *MYCN* amplification tested by FISH (*MYCN* FISH^+^), which was the maximum count of *MYCN* FISH^+^ prechemotherapy samples suitable for the IHC test during this time. As a control, 25 patients with *MYCN* FISH^−^ tumors were chosen according to their clinical consequences: 1) 8 patients died from tumors, 2) 17 patients had a favorable long-term prognosis. Follow-up within this cohort was completed on December 31, 2019. To ensure prognostic accuracy for individuals, only 41 patients (including 16 with FISH^+^ tumors and 25 with FISH^−^ tumors) of this cohort diagnosed in or before 2016 were included when referred to the follow-up time. More detailed clinical information was listed in Table 1 and Table S1.

**Table 1:** Key characteristics of the patient cohort

Characteristics	FISH^**−**^(***n*** = 25)	FISH^**+**^(***n*** = 28)	Total(***n*** = 53)
**IHC (MYCN)**			
0	20(37.74%)	4(7.55%)	24(45.28%)
1 ~ 8	4(7.55%)	6(11.32%)	10(18.87%)
≥9	1(1.89%)	18(33.96%)	19(35.85%)
**Age**			
> 18 m	13(24.53%)	19(35.85%)	32(60.38%)
≤18 m	12(22.64%)	9(16.98%)	21(39.62%)
**Stage**			
Stage 1	5(9.43%)	2(3.77%)	7(13.21%)
Stage 2	2(3.77%)	0(0.0e+ 0%)	2(3.77%)
Stage 3	8(15.10%)	12(22.64%)	20(37.74%)
Stage 4	7(13.21%)	14(26.42%)	21(39.62%)
Stage 4S	3(5.66%)	0(0.0e+ 0%)	3(5.66%)
**Risk**			
Low	6(11.32%)	0(0.0e+ 0%)	6(11.32%)
Med	7(13.21%)	3(5.66%)	10(18.87%)
High	5(9.43%)	14(26.42%)	19(35.85%)
Very High	7(13.21%)	11(20.75%)	18(33.96%)
**Complete primary tumor resection**			
No	5(9.43%)	6(11.32%)	11(20.75%)
Yes	20(37.74%)	22(41.51%)	42(79.25%)
**Autologous stem cell transplantation**			
No	21(39.62%)	26(49.06%)	47(88.68%)
Yes	4(7.55%)	2(3.77%)	6(11.32%)
**External Radiotherapy**			
No	18(33.96%)	13(24.53%)	31(58.49%)
Yes	7(13.21%)	15(28.30%)	22(41.51%)
**Event**			
No	17(32.08%)	19(35.85%)	36(67.92%)
Yes	8(15.09%)	9(16.98%)	17(32.08%)
**EFS months**			
Median [min-max]	58.43[4.70,109.73]	13.52[0.33,74.17]	28.00[0.33,109.73]
**Follow up status**			
CR	17(32.08%)	21(39.62%)	38(71.70%)
Death	8(15.09%)	7(13.21%)	15(28.30%)
**OS months**			
Median [min-max]	58.43[5.93,109.73]	16.73[0.33,74.17]	30.47[0.33,109.73]

Another two cohorts of 71 and 127 patients were identified as the validation cohorts for FISH and IHC results, respectively. Diagnostic tumor samples from the cohort of 71 patients were tested by whole exome sequencing (WES) and FISH at the same time, and those from the cohort of 127 patients were tested by MYCN IHC (MYCN antibody: # 51705, Cell Signaling Technology) and FISH at the same time. Because their other clinical information was not involved in this study, we would not further enumerate them.

### *MYCN* gene status tested by FISH

**Fig. 1:**
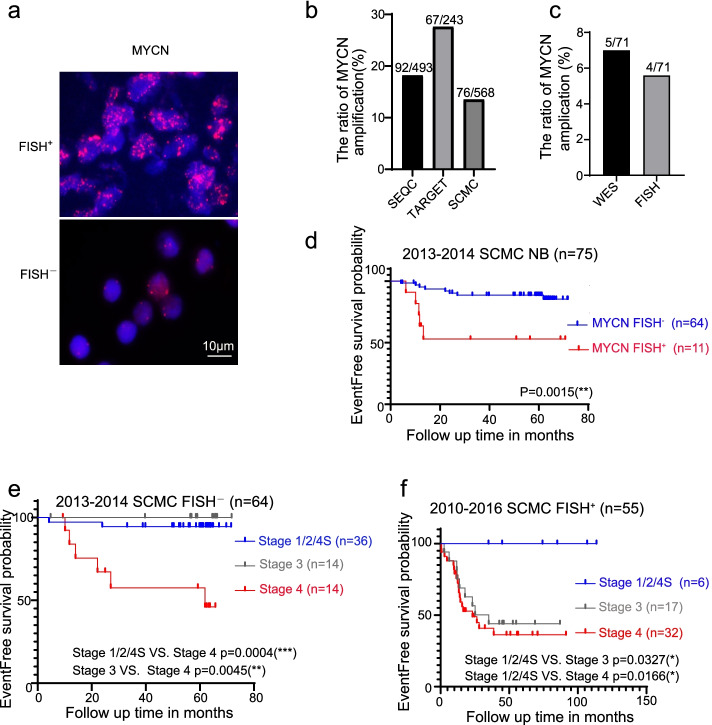
Identifying *MYCN* amplification status is most valuable for INSS stage 3 patients. **a** Representative *MYCN* FISH images of tumors from *MYCN* FISH^+^ and FISH^−^ samples (Red: *MYCN* region probe). **b** The ratio of *MYCN* amplification among SEQC, TARGET, and SCMC Dataset. **c** Comparing *MYCN* amplified rates detected by WES and FISH at SCMC. **d** Survival curve analysis of EFS cut off by *MYCN* amplification status. Log-rank (Mantel-Cox) test was used to generate the *p*-value. **e-f** Survival curve analysis of EFS cut off by INSS stages in *MYCN* FISH^−^ group (**e**) and *MYCN* FISH^+^ group (**f**). Log-rank (Mantel-Cox) test was used to generate the p-value

All 53 samples were evaluated *MYCN* amplification status by FISH using 2 μm formalin-fixed, paraffin-embedded (FFPE) sections. Laboratory-developed probes targeting *MYCN* gene (2p24) were used. Tissue sections were washed with SSC buffer and mounted in 4′, 6-diamidino-2-phenylindole for nuclear counterstaining. The results were analyzed and interpreted following the probe manufacturer’s instructions. *MYCN* FISH^+^ at region 2p24 showed red signals (Fig. 1a). If the copy numbers of *MYCN* were ≥ 5 per haploid genome, related patients were classified into the “*MYCN* FISH^+^” group.

### MYCN protein expression tested by IHC

Tumor specimens were fixed in 10% formalin and embedded in paraffin as soon as they were obtained from patients. Pathologists chose specimens with the highest tumor content by H&E staining to navigate tumor pathological heterogenicity. MYCN IHC was performed on the same specimen in which *MYCN* FISH was performed or on a different specimen of the same tumor if that was unavailable. The performance of two anti-MYCN antibodies (#84406 s and # 51705, Cell Signaling Technology) in IHC was compared. Subcutaneous tumors of *MYCN* amplified SK-N-BE(2) cell line were used as positive controls, and subcutaneous tumors of *MYCN* non-amplified SY-5Y cell line as negative controls. Scores for staining intensity were graded on a scale of 0–3 (0 = negative, 1 = weak, 2 = moderate, 3 = strong), while the positive proportion was graded on a scale of 0–4 (0 for 0%, 1 for < 25%, 2 for 25–50%, 3 for 50–75%, 4 for 75–100%). The IHC score was calculated independently by two pathologists blinded to the FISH results, based on the formula that final score = staining intensity * positive proportion. Samples with scores of 0 were defined as “IHC = 0”. Samples with scores of 1–9 were classified into the “low expression” group. Beyond that, samples belonged to the “high expression” group.

### RNA sequence

Total RNA was extracted and purified using the Qiagen RNeasy Mini kit (Valencia, CA, USA) according to the manufacturer’s instructions. The quality of RNA was assessed by a bioanalyzer before sequencing [20]. RNA libraries for RNA-seq were based on TruSeq Stranded Total RNA Gold library by Novaseq S4 PE150 (Illumina). Regrettably, only 14/53 samples detected by MYCN IHC had frozen tumor tissues and had been fully sequenced.

### Western blotting (WB)

WB was performed as previously described [21] against the following antibodies: rabbit anti-MYCN antibody (1:1000) (#84406 s, Cell Signaling Technology), mouse anti-β-actin antibody (1:10000) (HF1024, HuaAn).

### Statistical analysis

Data were processed with GraphPad Prism 8.0. An unpaired T-test was used to determine the statistical difference between groups. The time of event-free survival (EFS) was calculated from diagnosis until an event such as death, relapse, or progression; if there was no event, the date of last follow-up. The OS time was from diagnosis to death or the date of last follow-up. Kaplan-Meier EFS and OS analyses were performed using GraphPad Prism 8.0, and comparisons of survival curves were carried out using the log-rank (Mantel-Cox) test. A *p*-value < 0.05 was considered statistically significant.

### Data availability

The accession numbers of RNA-seq data and clinical information reported in this paper were SEQC Dataset (GSE62564) from the GEO website and TARGET Dataset deposited at the cancer genome atlas website.

## Results

### Identifying *MYCN* amplification status is most valuable for INSS stage 3 patients

As *MYCN* amplification closely correlates with the neoplastic prognosis of neuroblastoma [4], identifying *MYCN* amplification status is greatly important for related patients. The ratios of *MYCN* amplification among the SEQC dataset (*n* = 493), TARGET dataset (*n* = 243) and SCMC dataset (*n* = 568) were compared. Among the results, ratios in the SEQC dataset (*n* = 18.7%) and TAGET dataset (*n* = 27.6%) were within the universally acknowledged positive rate [22], while the ratio in the SCMC dataset (*n* = 13.4%) was far below (Fig. 1b). WES was performed to test the accuracy of SCMC FISH results. The results showed that the positive rates were almost equal between two methods (Fig. 1c), which meant that the FISH results in our hospital were reliable.

Then, patients diagnosed at SCMC during 2013–2014 were further analyzed. The prognosis of patients with *MYCN* FISH^+^ tumors was poor (*p* < 0.05, Kaplan-Meier survival analysis) (Fig. 1d). By categorizing patients with *MYCN* FISH^−^ tumors according to INSS stage, we found that the adverse event rate significantly increased when patients progressed to stage 4 (Fig. 1e). However, for patients with *MYCN* FISH^+^ tumors, their prognosis was poor once they developed to stage 3 (Fig. 1f). These results suggested that stage 1/2 and 4S patients usually had a promising future after rational treatment. If stage 3 patients with *MYCN* FISH^+^ tumors or stage 4 patients were older than 18 months at first diagnosis, their prognosis could hardly be sanguine even under the most aggressive treatment. Similar trends were found in database analysis (Supplemental Fig. 1). Overall, the *MYCN* gene test is most valuable for INSS stage 3 patients in predicting prognosis.

### MYCN IHC showed good specificity and sensitivity

Whether FISH or WES, both focus on *MYCN* gene status, while protein is the real one performing biological functions. However, there is no reliable antibody that could be clinically useful in IHC. Pathologists at SCMC tried various MYCN antibodies for a long time and finally chose one (MYCN antibody: #51705, Cell Signaling Technology) used in IHC. Between 2010 and 2015, 127 tumor samples were detected MYCN amplification status simultaneously by FISH and IHC (MYCN antibody: #51705, Cell Signaling Technology) at SCMC. Compared with FISH data, IHC results only had 43.1% concordance (50/116) in the *MYCN* FISH^−^ group and 36.4% concordance (4/11) in the *MYCN* FISH^+^ group (Table S2), which meant its specificity and sensitivity were substandard. Finding a reliable antibody that could rapidly and accurately detect MYCN protein expression was urgent. An antibody (#84406 s, Cell Signaling Technology), never used in the IHC test before, was chosen.

**Fig. 2:**
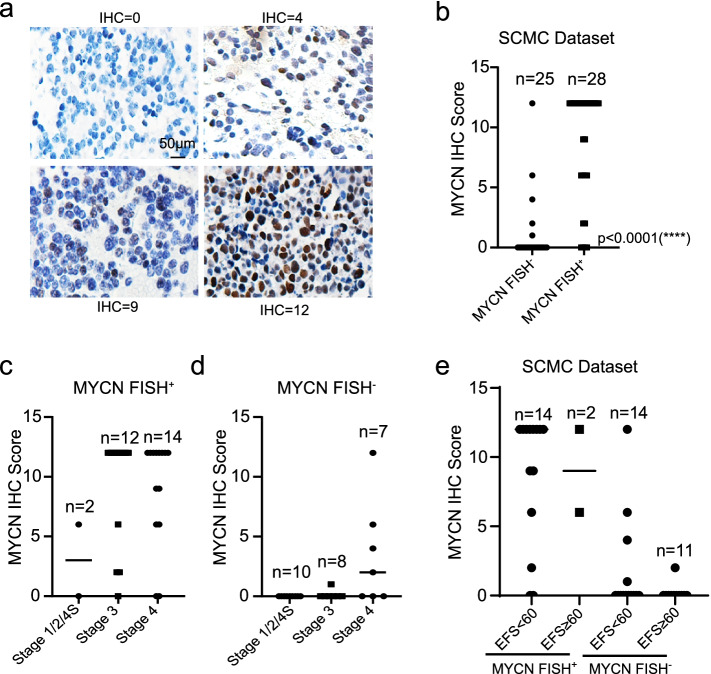
MYCN IHC showed good specificity and sensitivity. **a** Representative IHC images of tumors with different levels of MYCN protein expression (Immunostaining with hematoxylin counterstaining, Original*40). **b** The concordance of IHC and FISH about MYCN amplification status. An unpaired T-test was used to generate the *p*-value. **c-e** The IHC score of individuals distributed at different INSS stages and clinical outcomes

53 prechemotherapy samples were obtained, and 28 were *MYCN* FISH^+^ (Table 1). IHC detection revealed that their staining intensity and positive proportion of malignant cells showed a remarkable difference (Fig. 2a). With FISH results as standard, MYCN IHC could accurately detect more than 80% of cases regardless of whether the *MYCN* gene was amplified (Table 2, Fig. 2b). For those cases with inconsistent results, prognostic information was involved to verify which method was more reliable. The results showed that IHC scores increased with INSS stage (Fig. 2c, d). In the *MYCN* FISH^+^ group, 7/11 patients with high MYCN protein expression (IHC score ≥ 9) had a poor prognosis, whereas 5/5 with no or low (IHC score < 9) recovered well. In the *MYCN* FISH^−^ group, MYCN protein was not detected in 17/17 event-free patients’ tumors, but it was positive for 5/8 patients who died of neuroblastoma (Table 3-4, Fig. 2e). These data suggest that this MYCN antibody (#84406 s) has reasonable specificity and sensitivity.

**Table 2:** Inter-assay concordance analysis of MYCN amplification status determined by IHC and FISH in neuroblastoma

FISH	IHC = 0	0 < IHC < 9	IHC ≥ 9	Concordance by FISH	Discordance by FISH
FISH^−^(*n* = 25)	20(80.0%)	4(16.0%)	1(4.0%)	80.0%	20.0%
FISH^+^(*n* = 28)	4(14.3%)	6(21.4%)	18(64.3%)	85.7%	14.3%

**Table 3:** Inter-assay concordance analysis of MYCN protein expression with clinical consequences

NB	FISH^+^(*n* = 16)	IHC = 0(*n* = 2)	0 < IHC < 9(*n* = 3)	IHC ≥ 9(*n* = 11)
Event-free	9(56.3%)	2(100.0%)	3(100.0%)	4(36.4%)
Event	7(43.8%)	0(0.0%)	0(0.0%)	7(63.6%)
NB	FISH^−^(*n* = 25)	IHC = 0(*n* = 20)	0 < IHC < 9(*n* = 4)	IHC ≥ 9(*n* = 1)
Event-free	17(68.0%)	17(85.0%)	0(0.0%)	0(0.0%)
Event	8(32.0%)	3(15.0%)	4(100.0%)	1(100.0%)

**Table 4:** Inter-assay concordance analysis of MYCN stability with clinical consequences

NB	FISH^−^&IHC = 0 (*n* = 20)	FISH^−^&IHC > 0 (*n* = 5)	FISH^+^&IHC < 9 (*n* = 5)	FISH^+^&IHC ≥ 9 (*n* = 11)
CR	17(85.0%)	0(0.0%)	5(100.0%)	**6(54.5%)**
Death	3(15.0%)	5(100.0%)	0(0.0%)	5(45.5%)
Event-Free	17(85.0%)	0(0%)	5(100%)	**4(36.4%)**
Event	3(15.0%)	5(100%)	0(0%)	7(63.6%)

### Conjoint analysis of IHC and FISH provides better prognostic prediction

**Fig. 3:**
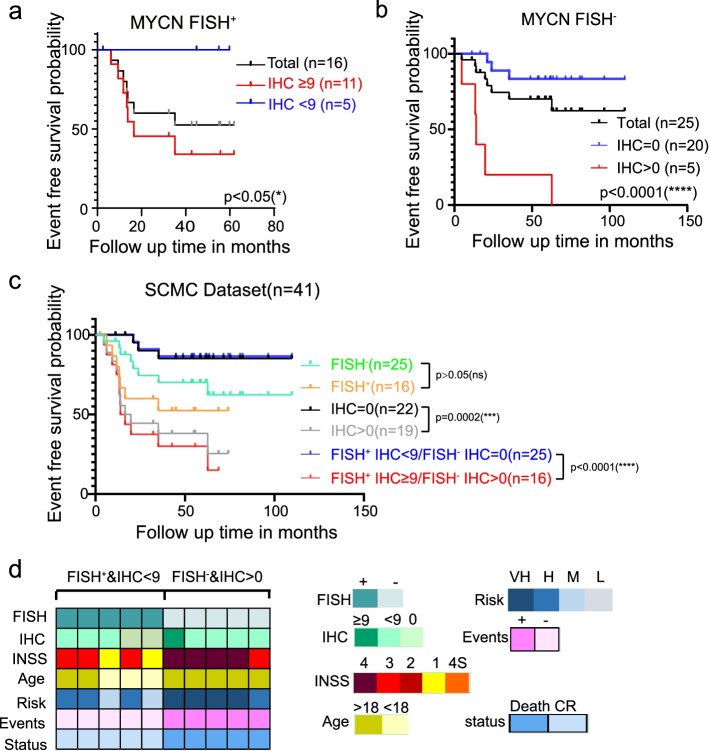
Conjoint analysis of IHC and FISH provides better prognostic prediction. **a-b** Survival curve analysis of EFS cut off by IHC score in the *MYCN* FISH^+^ group (*n* = 16) (**a**) and *MYCN* FISH^−^ group (*n* = 25) (**b**). Log-rank (Mantel-Cox) test was used to generate the p-value. To ensure prognostic accuracy for individuals, only 41 patients (including 16 with *MYCN* FISH^+^ tumors and 25 with *MYCN* FISH^−^ tumors) diagnosed in or before 2016 were included from the cohort of 53 patients. **c** Survival curve analysis of EFS cut off by IHC and FISH results (*n* = 41). Log-rank (Mantel-Cox) test was used to generate the p-value. Sample selection was the same with 3a-b. **d** The chromaticity diagram of several significant clinical factors

More analyses were performed to detect the value of MYCN IHC in clinical prediction. The results showed that IHC could further distinguish patients with different clinical outcomes in the *MYCN* FISH^+^ and FISH^−^ groups (Fig. 3a-b). If specifying patients only by FISH results, the *MYCN* FISH^+^ and FISH^−^ groups’ EFS rates were 56.3 and 68.0%, respectively. There was no significant difference (*p* > 0.05, Kaplan-Meier survival analysis). Grouped according to IHC results did better, and their difference was statistically significant (*p* < 0.05, Kaplan-Meier survival analysis). Combining IHC and FISH reached the best predicting effect among the three methods (Fig. 3c). As shown in Fig. 3d and Table 4, patients with *MYCN* FISH^−^ but protein-expressing tumors always had a poor prognosis. In contrast, patients with *MYCN* FISH^+^ but low protein expression tumors always had a favorable prognosis (Fig. 3d, Table 4). In summary, our results suggest that IHC could make up for FISH in predicting prognosis.

### MYCN protein stability is crucial for the prognosis of neuroblastoma

**Fig. 4:**
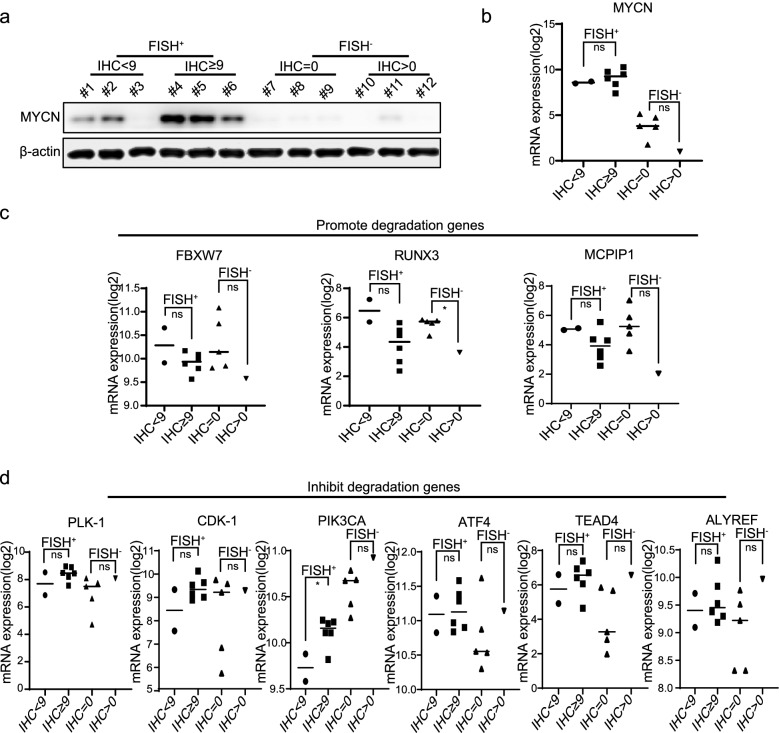
MYCN protein stability is crucial for the prognosis of neuroblastoma. **a** The MYCN protein expression tested by western blotting among 12 samples at SCMC. **b-d** The mRNA expression of *MYCN* and genes related to MYCN protein expression grouped by MYCN FISH and IHC results. An unpaired T-test was used to generate the p-value (* *p* < 0.05, ns: no significant)

FISH focused on DNA copy numbers, while IHC centered on protein expression. We wondered what exactly affected MYCN protein expression. Western blotting was performed to test MYCN protein expression (Fig. 4a). As predicted, their protein level varied from DNA copy numbers. For instance, the #3 tissue detected no protein expression but high DNA copy numbers. Based on RNA-seq data, we found that their *MYCN* mRNA expression was more consistent with the FISH results (Fig. 4b). These data demonstrated that aberrant protein expression might not stem from DNA copy numbers but be related to RNA translation or protein stability. Previous studies have shown that FBW7 [23], RUNX3 [24], and MCPIP1 [25] play a fundamental role in MYCN ubiquitination and degradation, and all these genes showed declining trends in the high MYCN protein expression group (Fig. 4c) (ns: no significant, unpaired T-test). PLK-1 [26], CDK-1 [27], PIK3CA [28], ATF4 [23], TEAD4 [29] and ALYREF [30] could enhance MYCN protein stability and sustain MYCN expression in neuroblastoma. The expression of these genes showed a rising trend in the high MYCN protein expression group (Fig. 4d) (* *p* < 0.05, ns: no significant, unpaired T-test). More factors that could affect MYCN protein expression were analyzed. The results showed that their expression was more consistent with the MYCN protein level detected by IHC (Fig. S2). Overall, some genes could influence MYCN protein expression through post-transcriptional or post-translational modification. RNA-seq revealed that their expression changed among different samples, which might be the reason why the results of MYCN IHC could be different from those of FISH. Conjoint analysis of IHC and FISH could test MYCN protein stability, a key prognostic factor.

## Discussion

MYCN is an identified driver and reliable genomic hallmark of aggressive tumor behavior [16]. Detecting *MYCN* amplification status has great significance for clinical treatment and prognostic prediction. This article clarified that identifying *MYCN* amplification status is most valuable for INSS stage 3 patients. MYCN stability detected by the joint of IHC and FISH is a good choice to predict the prognosis of neuroblastoma.

In clinical management, *MYCN* amplification status is a metric used to identify risk groups and determine chemotherapy regimens. FISH focuses on the *MYCN* DNA level; its result is regarded as the “gold standard” in clinical practice [11, 31]. Patients with *MYCN* FISH^+^ tumors would be classified into a higher risk group and given a higher dose of chemotherapy (Table S3). An ultrasensitive quantitative RNA in situ hybridization technique, RNA scope, is emerging [32]. This method investigates *MYCN* amplification status at the RNA level. Compared with CISH at the DNA level, it better predicts prognosis [32]. It is reasonable to suspect that identifying MYCN protein levels is the best choice to assess prognosis among the above.

However, most hospitals have to give up MYCN IHC for poor antibodies, so there is little research about the correlation between *MYCN* amplification status and protein expression. MYCN antibodies from Santa Cruz Biotechnology Inc. [11] or Abcam [17–19] are several antibodies mentioned in research, but high false-positive and false-negative rates were observed in IHC using the antibodies mentioned above in our hospital. In this study, we extended the application of a commercial MYCN antibody in IHC. Both specificity and sensitivity of this antibody showed bright futures. Compared with before, the accuracy of IHC was vastly improved. In addition, experimental results would be less affected by human factors with the application of IHC autostainer. For neuroblastoma patients, especially those without *MYCN* amplification but with abnormal protein expression, MYCN IHC has great clinical value. A joint of IHC and FISH could obtain a more complete understanding of MYCN expression level. It could reduce the shortcoming of any single method and obtain a better predictive effect. However, our research is limited by relatively small samples; we need more high-quality randomized controlled trials to provide more evidence.

In addition to *MYCN* gene status, age of diagnosis is an important indicator of risk stratification and is closely related to prognosis. Children younger than 12 months at first diagnosis may be classified to stage 4S, and they might resolve themselves whether the *MYCN* gene is amplified or not. The elucidation of the intrinsic mechanisms of stage 4S patients with a good prognosis could offer new ideas to cure neuroblastoma.

In summary, MYCN is a vital index influencing neuroblastoma prognosis. The combination of IHC and FISH to determine MYCN stability could potentially be of greater importance as prognostic indicators for patients diagnosed with neuroblastoma compared to singular factors.

## Supplementary Information

**Additional file 1: Supplemental Fig. 1.** a-d Survival curve analysis of EFS (a-b) and OS(c-d) when *MYCN* amplification (b,d) or not (a,c). Log-rank (Mantel-Cox) test was used to generate the *p*-value.

**Additional file 2: Supplemental Fig. 2.** a Heat map grouped by MYCN FISH and IHC results. The mRNA expression of *MYCN* and genes related to MYCN protein stability were shown.

**Additional file 3.**

**Additional file 4.**

**Additional file 5.**

## Data Availability

The data that support the findings of this study are available on request from the corresponding author. The data are not publicly available due to privacy or ethical restrictions.

## Abbreviations

FISH: Fluorescence in situ hybridization
OS: Overall survival
WES: Whole exome sequencing
PCR: Polymerase chain reaction
qPCR: Quantitative real-time PCR
SQ-PCR: Semi-quantitative differential PCR
ddPCR: Droplet digital PCR
CISH: Chromogenic in situ hybridization
MLPA: Multiplex ligation-dependent probe amplification
IHC: Immunohistochemistry
FFPE: Formalin-fixed, paraffin-embedded
VCR: Vincristine
CTX: Cyclophosphamide
CBP: Carboplatin
VP-16: Etoposide
Adr: Adriamycin
CDDP: Cisplatin
IFOS: Ifosfamide
THP: Pirarubicin
Topo: Topotecan
DOXO: Doxorubicin
