# Supplementary figures and images for: MYCN protein stability is a better prognostic indicator in neuroblastoma
Source: BMC Pediatr. 2022 Jul 11;22:404. doi: 10.1186/s12887-022-03449-1 (PMC9277955; doi:10.1186/s12887-022-03449-1)

Supplemental Figure 1

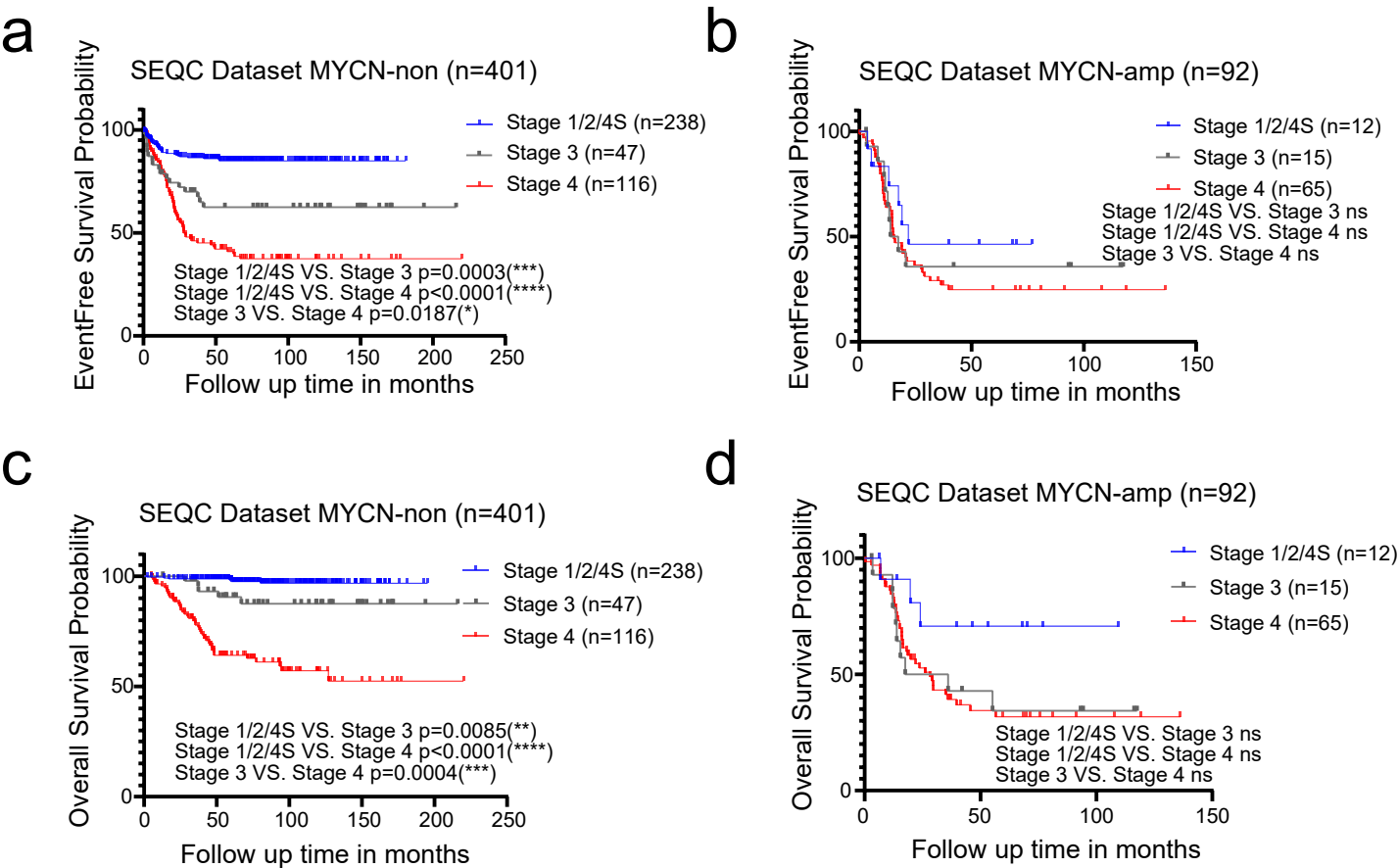

Supplement: Supplementary file 1 — Additional file 1: Supplemental Fig. 1. a-d Survival curve analysis of EFS (a-b) and OS(c-d) when MYCN amplification (b,d) or not (a,c). Log-rank (Mantel-Cox) test was used to generate the p-value. [file 12887_2022_3449_MOESM1_ESM.pdf]

Supplemental Figure 2

a

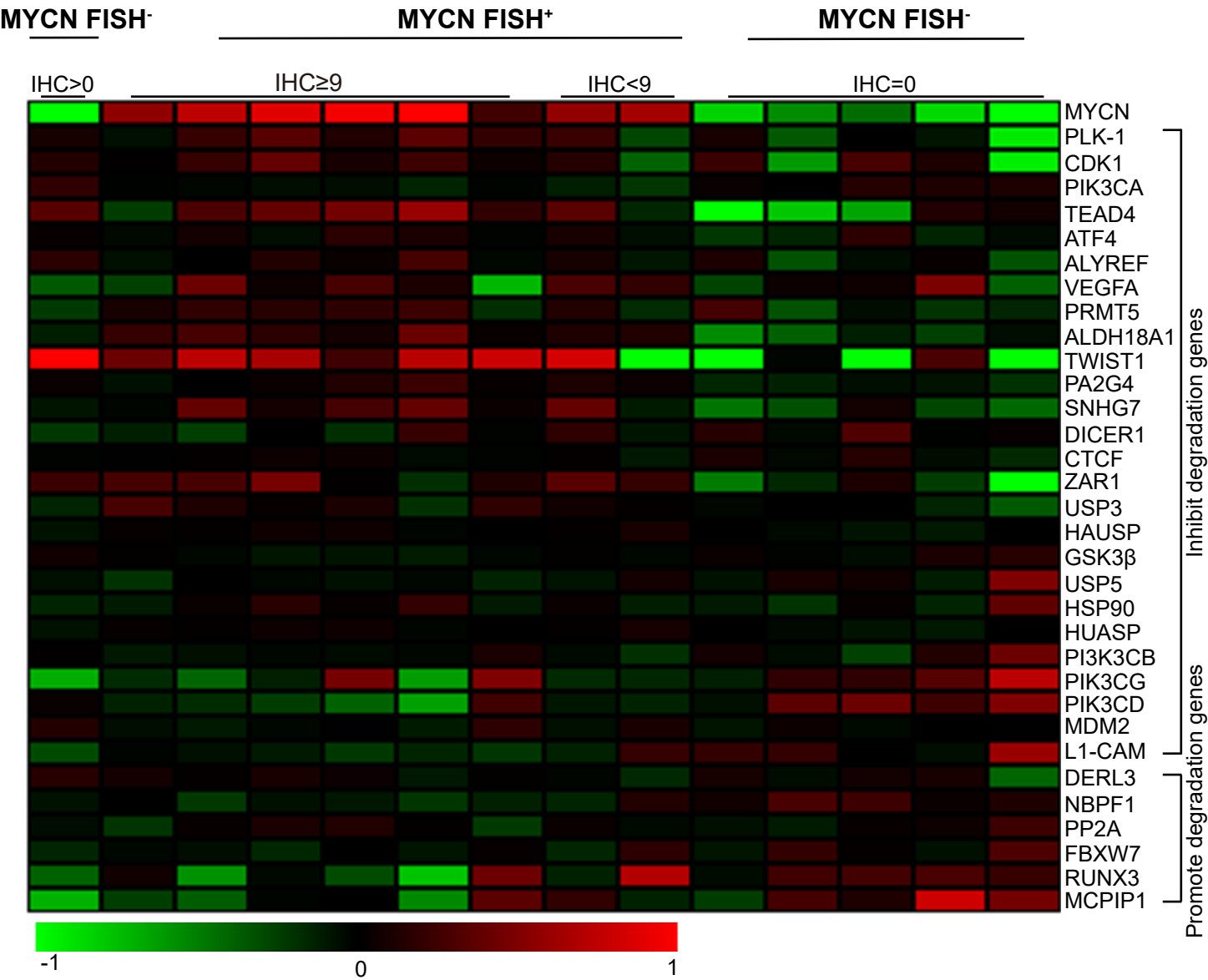

Supplement: Supplementary file 2 — Additional file 2: Supplemental Fig. 2. a Heat map grouped by MYCN FISH and IHC results. The mRNA expression of MYCN and genes related to MYCN protein stability were shown. [file 12887_2022_3449_MOESM2_ESM.pdf]
