# Supplementary material for: MYCN protein stability is a better prognostic indicator in neuroblastoma
Source: BMC Pediatr. 2022 Jul 11;22:404. doi: 10.1186/s12887-022-03449-1 (PMC9277955; doi:10.1186/s12887-022-03449-1)
Supplement: Supplementary file 3 — Additional file 3. [file 12887_2022_3449_MOESM3_ESM.docx]

| Table S1 Clinical data of patients | | | | | | | | | | | | | | | | | |
| --- | --- | --- | --- | --- | --- | --- | --- | --- | --- | --- | --- | --- | --- | --- | --- | --- | --- |
| **Case** | **Age** | **Stage** | **Risk** | **Primary Sites** | **Infiltrating Sites** | **FISH**  **(MYCN)** | **IHC (MYCN)** | **Distant Transference** | **Therapy protocol** | **Course of Therapy** | **Complete primary tumor resection** | **autologous stem cell transplantation** | **Radio-therapy** | **Event** | **EFS months** | **OS months** | **Follow up status** |
| 1 | 2.90 | 4 | 4 | 3 | 5 | 5 | 6 | 1 | 2016Very High Risk | 9 | Yes | No | No | Relapse | 15.23 | 16.30 | Death |
| 2 | 2.44 | 4 | 4 | 3 | 5+10 | 5 | 6 | 1 | 2016Very High Risk | 10 | Yes | No | Yes |  | 8.10 | 8.10 | CR |
| 3 | 1.87 | 3 | 3 | 3 |  | 5 | 2 | 0 | 2016High Risk | 8 | Yes | No | No |  | 10.50 | 10.50 | CR |
| 4 | 1.81 | 4 | 4 | 3 | 5+6+12 | 5 | 9 | 1 | 2010Very High Risk | 11 | Yes | No | Yes | Progress | 16.53 | 18.93 | CR |
| 5 | 3.63 | 3 | 3 | 3 |  | 5 | 12 | 0 | 2016High Risk | 7 | No | No | Yes |  | 27.07 | 27.07 | CR |
| 6 | 1.38 | 3 | 3 | 3 |  | 5 | 12 | 0 | 2010High Risk | 6 | Yes | No | No | Progress | 35.10 | 53.93 | Death |
| 7 | 4.08 | 4 | 4 | 3 | 5+6 | 1 | 0 | 1 | 2010Very High Risk | 10 | No | Yes | Yes | Relapse | 35.30 | 38.33 | Death |
| 8 | 7.11 | 4 | 4 | 3 | 5 | 1 | 0 | 1 | 2016Very High Risk | 8 | Yes | No | Yes | Relapse | 23.97 | 24.03 | Death |
| 9 | 0.27 | 1 | 1 | 2 |  | 1 | 0 | 0 |  |  | Yes | No | No |  | 62.10 | 62.10 | CR |
| 10 | 0.16 | 2 | 1 | 3 | 10 | 1 | 0 | 0 |  |  | Yes | No | No |  | 75.33 | 75.33 | CR |
| 11 | 0.09 | 4S | 2 | 3+4 | 9 | 1 | 0 | 1 | 2010Med Risk | 7 | Yes | No | No |  | 71.53 | 71.53 | CR |
| 12 | 0.21 | 1 | 1 | 2 |  | 1 | 0 | 0 |  |  | Yes | No | No |  | 109.73 | 109.73 | CR |
| 13 | 11.96 | 3 | 3 | 3 |  | 1 | 0 | 0 | 2010High Risk | 4 | Yes | No | No |  | 82.20 | 82.20 | CR |
| 14 | 2.98 | 3 | 3 | 1 |  | 1 | 0 | 0 | 2010High Risk | 8 | Yes | No | Yes |  | 80.90 | 80.90 | CR |
| 15 | 0.34 | 1 | 1 | 3 |  | 1 | 0 | 0 |  |  | Yes | No | No |  | 65.87 | 65.87 | CR |
| 16 | 3.27 | 3 | 3 | 3 |  | 1 | 0 | 0 | 2010High Risk | 6 | Yes | No | Yes |  | 96.57 | 96.57 | CR |
| 17 | 3.01 | 4 | 4 | 3 | 5+7+12 | 1 | 4 | 1 | 2010Very High Risk | 10 | No | No | Yes | Relapse | 19.80 | 20.63 | Death |
| 18 | 1.58 | 3 | 3 | 3 |  | 5 | 2 | 0 | 2010High Risk | 6 | No | No | Yes |  | 55.03 | 55.03 | CR |
| 19 | 6.59 | 3 | 3 | 3 |  | 5 | 6 | 0 | 2008High Risk | 3 | No | No | No |  | 2.57 | 2.57 | CR |
| 20 | 1.29 | 4 | 3 | 3 | 8 | 5 | 12 | 1 | 2010High Risk | 9 | No | No | Yes | Relapse | 9.40 | 9.40 | Death |
| 21 | 3.75 | 4 | 4 | 3 | 5 | 1 | 2 | 1 | 2010Very High Risk | 10 | No | Yes | Yes | Relapse | 62.67 | 63.13 | Death |
| 22 | 1.18 | 3 | 2 | 3+4 | 7+10 | 1 | 0 | 0 | 2010Med Risk | 8 | Yes | No | No |  | 61.57 | 61.57 | CR |
| 23 | 3.07 | 3 | 3 | 3 |  | 5 | 12 | 0 | 2010High Risk | 7 | Yes | No | Yes |  | 42.70 | 42.70 | CR |
| 24 | 3.81 | 3 | 3 | 3 | 9+17 | 1 | 1 | 0 | 2010High Risk | 10 | Yes | No | No | Death | 13.37 | 13.83 | Death |
| 25 | 1.01 | 1 | 2 | 3 |  | 5 | 6 | 0 | 2010Med Risk | 4 | Yes | No | No |  | 74.17 | 74.17 | CR |
| 26 | 1.21 | 3 | 3 | 3+4 |  | 5 | 12 | 0 | 2010High Risk | 8 | Yes | Yes | No |  | 68.77 | 68.77 | CR |
| 27 | 2.86 | 3 | 3 | 3 |  | 5 | 12 | 0 | 2010High Risk | 10 | No | No | Yes | Relapse | 11.77 | 11.77 | Death |
| 28 | 3.30 | 2 | 2 | 2 |  | 1 | 0 | 0 | 2010Med Risk | 6 | Yes | No | No |  | 63.50 | 63.50 | CR |
| 29 | 3.98 | 4 | 4 | 3 | 10 | 5 | 9 | 1 | 2010Very High Risk | 10 | Yes | No | Yes | Relapse | 13.27 | 13.27 | Death |
| 30 | 1.68 | 3 | 3 | 3 |  | 5 | 12 | 0 | 2010High Risk | 9 | Yes | No | Yes | Relapse | 6.17 | 14.13 | Death |
| 31 | 2.46 | 4 | 4 | 3 | 5+6 | 5 | 12 | 1 | 2010Very High Risk | 10 | Yes | No | No |  | 32.33 | 32.33 | CR |
| 32 | 2.52 | 4 | 4 | 2+3 | 5 | 1 | 6 | 1 | 2010Very High Risk | 10 | Yes | No | No | Relapse | 13.97 | 17.30 | Death |
| 33 | 0.59 | 1 | 1 | 2 |  | 1 | 0 | 0 |  |  | Yes | No | No |  | 58.43 | 58.43 | CR |
| 34 | 3.74 | 4 | 4 | 3 | 5+6 | 5 | 12 | 1 | 2010Very High Risk | 10 | Yes | No | Yes |  | 55.70 | 55.70 | CR |
| 35 | 0.23 | 3 | 2 | 1 |  | 1 | 0 | 0 | 2010Med Risk | 6 | Yes | No | No |  | 54.37 | 54.37 | CR |
| 36 | 0.38 | 4S | 2 | 3 | 9+10 | 1 | 0 | 1 | 2010Med Risk | 8 | Yes | No | No |  | 48.93 | 48.93 | CR |
| 37 | 3.33 | 4 | 4 | 3 | 5+6+7 | 1 | 0 | 1 | 2010Very High Risk | 10 | No | Yes | No |  | 53.67 | 53.67 | CR |
| 38 | 0.35 | 4S | 2 | 3 | 9 | 1 | 0 | 1 | 2010Med Risk | 8 | Yes | No | No |  | 58.80 | 58.80 | CR |
| 39 | 3.83 | 3 | 3 | 3 |  | 1 | 0 | 0 | 2016High Risk | 8 | Yes | No | Yes | Relapse | 21.13 | 30.47 | Death |
| 40 | 1.53 | 4 | 4 | 3 | 9 | 1 | 12 | 1 | 2016Very High Risk | 6 | Yes | Yes | No | Relapse | 4.70 | 5.93 | Death |
| 41 | 0.34 | 3 | 2 | 3 |  | 1 | 0 | 0 | 2016Med Risk | 6 | No | No | No |  | 16.63 | 16.63 | CR |
| 42 | 2.13 | 3 | 3 | 3 |  | 5 | 12 | 0 | 2016High Risk | 8 | No | Yes | Yes | Relapse | 13.77 | 20.17 | CR |
| 43 | 0.96 | 3 | 3 | 4 |  | 5 | 0 | 0 | 2016High Risk | 8 | Yes | No | No |  | 44.97 | 44.97 | CR |
| 44 | 0.34 | 1 | 2 | 3 |  | 5 | 0 | 0 | 2016Med Risk | 4 | Yes | No | No |  | 44.87 | 44.87 | CR |
| 45 | 0.22 | 1 | 1 | 2 |  | 1 | 0 | 0 |  |  | Yes | No | No |  | 11.23 | 11.23 | CR |
| 46 | 1.22 | 4 | 3 | 3 | 6 | 5 | 12 | 1 | 2016High Risk | 10 | Yes | No | Yes |  | 28.00 | 28.00 | CR |
| 47 | 0.50 | 4 | 2 | 3 |  | 5 | 12 | 1 | 2016Med Risk |  | Yes | No | No |  | 0.33 | 0.33 | CR |
| 48 | 3.94 | 4 | 4 | 3 |  | 5 | 12 | 1 | 2016Very High Risk | 10 | Yes | No | Yes |  | 12.77 | 12.77 | CR |
| 49 | 4.75 | 4 | 4 | 3 | 5+6 | 5 | 0 | 1 | 2016Very High Risk | 10 | Yes | No | No |  | 11.47 | 11.47 | CR |
| 50 | 3.41 | 4 | 4 | 3 | 5+6+7+9 | 5 | 0 | 1 | 2016Very High Risk | 10 | Yes | No | Yes | Relapse | 9.47 | 17.17 | Death |
| 51 | 1.50 | 4 | 4 | 3 | 5 | 5 | 12 | 1 | 2016Very High Risk | 10 | Yes | No | No |  | 7.23 | 7.23 | CR |
| 52 | 4.11 | 3 | 3 | 3 |  | 5 | 12 | 0 | 2016High Risk | 10 | Yes | No | Yes |  | 6.50 | 6.50 | CR |
| 53 | 1.89 | 4 | 4 | 3 | 5+6 | 5 | 12 | 1 | 2016Very High Risk | 8 | Yes | No | No |  | 4.20 | 4.20 | CR |

Risk: 1 low risk; 2 med risk; 3 high risk; 4 very-high risk

Primary sites: 1 cervical part; 2 thorax; 3 abdomen; 4 pelvic cavity

Infiltrating sites: 5 distant bone; 6 bone marrow; 7 craniocerebrum; 8 soft tissue; 9 liver; 10 distant lymph node; 11 intraspinal canals; 12 hydrothorax; 13 breast; 14 testis; 15 lung; 16 spleen; 17 pancreas;
