# Supplementary material for: MYCN protein stability is a better prognostic indicator in neuroblastoma
Source: BMC Pediatr. 2022 Jul 11;22:404. doi: 10.1186/s12887-022-03449-1 (PMC9277955; doi:10.1186/s12887-022-03449-1)
Supplement: Supplementary file 4 — Additional file 4. [file 12887_2022_3449_MOESM4_ESM.doc]

Supplemental Table 2 Inter-assay concordance analysis of MYCN status determined by IHC and FISH in human neuroblastoma during 2010-2015 at SCMC with another MYCN antibody (MYCN antibody：# 51705, Cell Signaling Technology ).

|  | **IHC (-)** | **IHC(-/+)** | **IHC(+)** | **Total** | **Concordance** |
| --- | --- | --- | --- | --- | --- |
| **FISH^-^** | 50 | 15 | 51 | 116 | 43.1% |
| **FISH^+^** | 5 | 2 | 4 | 11 | 36.4% |
| **Tota**l | 55 | 17 | 55 | 127 | 42.5% |
