# Supplementary material for: MYCN protein stability is a better prognostic indicator in neuroblastoma
Source: BMC Pediatr. 2022 Jul 11;22:404. doi: 10.1186/s12887-022-03449-1 (PMC9277955; doi:10.1186/s12887-022-03449-1)
Supplement: Supplementary file 5 — Additional file 5. [file 12887_2022_3449_MOESM5_ESM.doc]

Supplemental Table 3 Risk classification scheme

| INSS | Age(month) | | | IDRFs  （diagnosis、before operation） | | MYCN | | Risk Group | |
| --- | --- | --- | --- | --- | --- | --- | --- | --- | --- |
|  | <12 | 12-18 | >18 | L1/L2 and VGPR after operation | L2 and  PR after operation | ≧5 per haploid genome | <5 per haploid genome |  |  |
| 1 | Yes | Yes | Yes | Yes | ***** | ***** | Yes | LOW Risk | Any*****is Med Risk |
| 2 | Yes | Yes | ***** | Yes | ***** | ***** | Yes | LOW Risk | Any*****is Med Risk |
| 3 | Yes | Yes | ***** | Yes | Yes | ***** | Yes | Med  Risk | Any*****is High Risk |
| 4 | Yes | Yes | ****** | any | any | ***** | Yes | Med  Risk | *****is High Risk、  ****** is Very-High Risk |
| 4S | Yes | Yes | -- | any | any | ***** | Yes | Med  Risk | ***** is High Risk |

IDRFs: Image-defined risk factors [33]

L1: no IDRFs L2: one or more IDRFs

**INSS staging system** [34]

**Stage 1:** Localized tumour with complete gross surgical excision and no metastasis to the representative ipsilateral lymph nodes that were not attached to tumour.

**Stage 2A**: Localized tumour with incomplete gross surgical excision and no metastasis to the lymph nodes.

**Stage 2B:** Localized tumour with or without complete gross surgical excision, with tumour metastasis to the ipsilateral lymph nodes but no tumour metastasis noted in any enlarged contralateral lymph nodes.

**Stage 3**: Unresectable, unilateral tumour infiltrating across the midline, with or without regional lymph node metastasis, or localized unilateral tumour with contralateral regional lymph node metastasis, or midline tumour with bilateral infiltration or lymph node involvement.

**Stage 4:** Any primary tumour with metastasis to distant lymph nodes and/or other organs, except as defined for stage 4S.

**Stage 4S**: Localized primary tumour (stages 1, 2A or 2B) in patients <1 year of age, with metastasis limited to the skin, liver or bone marrow (<10% tumour involvement).

**Treatments based on risk stratification**

| **Risk Group** | **Low** | **Med** | **High** | **Very High** |
| --- | --- | --- | --- | --- |
| **Treatment Principles** | Closely observation after Surgery | 4 courses subsequent to achieving VGPR, total <8 courses | 4 courses subsequent to achieving VGPR, total <10 courses | 6 courses subsequent to achieving VGPR, total <10 courses |
| **Chemotherapy Arrangement** | **-** | Course1,3,5,7:VCR+CBP+ADR+CTX➀  Course2,4,6,8:VCR+CBP+VP-16+CTX➀ | Course1,3,5,7,9:VCR+CDDP+VP16+CTX➁  Course2,4,6,8,10:IFOS+CBP+THP➁ | Course1,2,7,10:  CTX*+TOPO➂  Course3,5,8:  CDDP+VP-16➂  Course4,6,9:CTX+DOXO+VCR+MESNA➂ |
| **ABMT** | **-** | **-** | Heavy tumor burden with LDH 5 times higher than normal | YES |
| **Radiotherapy**  **Maintenance** | - | 13-cis-retinoic acid | Age>18 months  13-cis-retinoic acid | Age>18 months  13-cis-retinoic acid |

➀VCR:1.5mg/m2,d1; CTX:1.0g/m2,d1; CBP:550mg/m2,d2; VP-16:160mg/m2,d4; Adr: 30mg/ m2,d4.

➁VCR:1.5mg/m2,d1,8; CTX:1.0g/m2,d1,2; CDDP:25mg/m2,d1-5; VP-16:100mg/m2,d1-5; IFOS:1.5g/m2,d1-5; THP:30mg/ m2,.d1; CBP: 550mg/ m2,d2.

➂CTX*:400 mg/m2/d,d1-5; Topo: 1.2 mg/m2/d,d1-5; CDDP: 50 mg/m2/d,d1-4; VP-16: 200 mg/m2/d,d1-3 ; CTX: 1800 mg/m2/d,d1-2; Mesna:420 mg/m2/dose,q4hx3,d1-2; DOXO:25 mg/m2/d,d1-3; VCR:1.5 mg/m2/d,d1.
